# ACEA Attenuates Oxidative Stress by Promoting Mitophagy via CB1R/Nrf1/PINK1 Pathway after Subarachnoid Hemorrhage in Rats

**DOI:** 10.1155/2022/1024279

**Published:** 2022-02-24

**Authors:** Binbing Liu, Yang Tian, Yuchen Li, Pei Wu, Yongzhi Zhang, Jiaolin Zheng, Huaizhang Shi

**Affiliations:** ^1^Department of Neurosurgery, The First Affiliated Hospital of Harbin Medical University, Harbin, Heilongjiang, China; ^2^Department of Neurology, The Second Affiliated Hospital of Harbin Medical University, Harbin, Heilongjiang, China

## Abstract

**Method:**

Endovascular perforation was performed to establish a SAH model of rats. ACEA was administered intraperitoneally 1 h after SAH. The CB1R antagonist AM251 was injected intraperitoneally 1 h before SAH induction. Adenoassociated virus- (AAV-) Nrf1 shRNA was infused into the lateral ventricle 3 weeks before SAH induction. Neurological tests, immunofluorescence, DHE, TUNEL, Nissl staining, transmission electron microscopy (TEM), and Western blot were performed.

**Results:**

The expression of CB1R, Nrf1, PINK1, Parkin, and LC3II increased and peaked at 24 h after SAH. ACEA treatment exhibited the antioxidative stress and antiapoptosis effects after SAH. In addition, ACEA treatment increased the expression of Nrf1, PINK1, Parkin, LC3II, and Bcl-xl but repressed the expression of Romo-1, Bax, and cleaved caspase-3. Moreover, the TEM results demonstrated that ACEA promoted the formation of mitophagosome and maintained the normal mitochondrial morphology of neurons. The protective effect of ACEA was reversed by AM251 and Nrf1 shRNA, respectively.

**Conclusions:**

This study demonstrated that ACEA alleviated oxidative stress and neurological dysfunction by promoting mitophagy after SAH, at least in part via the CB1R/Nrf1/PINK1 signaling pathway.

## 1. Introduction

Subarachnoid hemorrhage (SAH) is a severe subtype of stroke with high morbidity and mortality [1]. Although the early diagnosis and treatment methods have been improved in the past few decades, not every patient can achieve a good clinical prognosis because of early brain injury (EBI) [2, 3]. Recent studies reported that oxidative stress plays a pivotal role in EBI after SAH [4–6]. Thus, alleviating oxidative stress injury may be an efficacious treatment for improving the prognosis of SAH.

Dysfunctional mitochondria are the primary source of intracellular reactive oxygen species (ROS) due to the disruption of the electron transfer chain and the transition of mitochondrial membrane permeability following SAH [4, 7], leading to oxidative stress injury and subsequent neuronal death [6]. Consequently, the clearance of impaired mitochondria in time would be an effective treatment strategy for SAH patients. Mitophagy is a selective autophagy process that specifically degrades damaged mitochondria to maintain mitochondrial homeostasis and cellular survival [8, 9]. Accumulating evidence has indicated that mitophagy is a potential therapeutic target to protect against EBI after SAH [10–13]. Furthermore, our previous research proved that promoting mitophagy alleviated oxidative stress as well as subsequent neuronal death after SAH [14].

The endocannabinoid system consists of lipid-based mediators, endocannabinoids (eCB), their target receptors, associated synthesizing and metabolizing enzymes, and transporter proteins. It is reported that the level of cannabinoid receptors and endocannabinoids increased after stroke [15]. Cannabinoid receptor 1 (CB1R) is a G-protein-coupled receptor, which is involved in modulation of neuronal activity, synaptic plasticity, and cell metabolism [16–18]. Accumulating evidence has demonstrated that activation of CB1R is beneficial to provide neuroprotection for stroke [19–21].

Nuclear respiratory factor 1 (Nrf-1) is a critical transcription factor that regulates genes necessary for mitochondrial biogenesis and function [22–24]. In recent years, it has been discovered that NRF1 target genes are not restricted to the genes involved in mitochondrial function, which indicates that Nrf-1 has more potential functions [25]. For example, Nrf-1 has a positive regulatory effect on the expression of PINK1 and Parkin genes, and it participates in mitochondrial quality control by regulating the PINK1/Parkin-mediated mitophagy [26]. Arachidonyl-2-chloroethylamide (ACEA), a highly selective CB1R agonist, was reported to protect neurons against ischemic injury by increasing the expression of Nrf1 and inducing mitochondrial biogenesis [27]. However, whether the protective effect of ACEA is mediated by regulating mitophagy remains unknown.

Thus, our study was aimed at verifying the hypothesis that ACEA attenuates oxidative stress by regulating mitophagy via the CB1R/Nrf1/PINK1 pathway after SAH in rats.

## 2. Materials and Methods

### 2.1. Animals and SAH Model

All experimental procedures were approved by the Institutional Animal Care and Use Committees of the First Affiliated Hospital of Harbin Medical University and were in accordance with the NIH Guidelines for the Care and Use of Laboratory Animals. Adult male Sprague-Dawley rats (weight 280-320 g) were housed at a constant humidity (55 ± 5%) and temperature (22 ± 2°C) in a 12 h light and dark cycle room. The animals were raised with free access to food and water.

The endovascular perforation method was employed to induce a SAH model in rats, as previously described [28]. Briefly, after rats were fully anesthetized, the external carotid artery (ECA) and internal carotid artery (ICA) were fully exposed. A sharp 4–0 nylon suture was inserted into the left ICA from the cut of the ECA stump until resistance was felt. The suture was further advanced to puncture the artery for several seconds, then withdrawn immediately. In Sham rats, all procedures were identical except the puncture of the vessel.

### 2.2. Drug Administration

The highly selective CB1R agonist ACEA (Cat. No.1319, Tocris Bioscience, Bristol, UK) was diluted in 5% dimethyl sulfoxide (DMSO). ACEA was administered intraperitoneally (i.p.) in different groups at doses of 0.5 mg/kg, 1.5 mg/kg, and 4.5 mg/kg 1 h after SAH [27]. The CB1R antagonist AM251 (Cat. No. A6226, Sigma-Aldrich, MO, USA), dissolved in 5% DMSO, was injected intraperitoneally at a dose of 1.0 mg/kg 1 h before SAH induction [29]. The control groups were injected the same volume of solvents, respectively.

### 2.3. Intracerebroventricular Injection

An adenoassociated virus (AAV; GeneChem, Shanghai, China) system was used to knock down the expression of Nrf1 according to the manufacturer's instructions. A nontargeting scrambled negative matched shRNA was used as a control. Animals were injected intraperitoneally with pentobarbital (40 mg/kg) and placed in a stereotaxic apparatus. Next, a 10 *μ*L syringe was inserted into the left ventricle at the specific coordinates relative to the bregma: 1.0 mm lateral, 1.5 mm posterior, and 3.1 mm below the dural surface. A total of 3 *μ*L of Nrf1 shRNA or scrambled shRNA was injected intraventricularly at a rate of 0.3 *μ*L/min in 3 weeks before SAH induction. To improve the knockdown efficiency, three different shRNA duplexes were designed and mixed. Their sequences are provided as follows: shRNA1: 5′-GCCTGGTCCAGATCCCTGTGACGAATCACAGGGATCTGGACCAGGCTTTTT-3′, shRNA2: 5′-GGACAGCGCAGTCACCATGGACGAATCCATGGTGACTGCGCTGTCCTTTTT-3′, and shRNA3: 5′-GGAGGTGGTGACGTTGGAACACGAATGTTCCAACGTCACCACCTCCTTTTT-3′.

### 2.4. Experimental Design (Supplemental Figure 1)

#### 2.4.1. Experiment 1

36 rats (*n* = 6 per group) were randomly assigned into 6 groups: Sham and 3, 6, 12, 24, and 72 hours, after SAH. Expression of CB1R, Nrf1, PINK1, Parkin, and LC3II/LC3I was analyzed by Western blot. Additionally, 3 Sham rats and 3 SAH-24 h rats were used to detect the cellular localization of CB1R by immunofluorescence staining.

#### 2.4.2. Experiment 2

In a short-term outcome assessment, 30 rats (*n* = 6 per group) were equally assigned into 5 groups: Sham, SAH+vehicle, SAH+ACEA (0.5 mg/kg), SAH+ACEA (1.5 mg/kg), and SAH+ACEA (4.5 mg/kg) for neurological tests. According to neurobehavioral test results, 1.5 mg/kg was chosen as the best dosage for subsequent experiments.

#### 2.4.3. Experiment 3

In a long-term outcome assessment, 18 rats (*n* = 6 per group) were assigned into 3 groups: Sham, SAH+vehicle, and SAH+ACEA. Rotarod test, foot fault test, and Nissl staining were performed.

#### 2.4.4. Experiment 4

To verify the neuroprotective effect of ACEA, 30 rats (*n* = 6 per group) were assigned into 5 groups: Sham, SAH+vehicle, SAH+ACEA, SAH+AM251, and SAH+ACEA+AM251 for Western blot. Additionally, 30 rats (*n* = 6 per group) were used for neurological tests, DHE, TUNEL, immunofluorescence staining, and transmission electron microscopy (TEM). Samples for determination of MDA, SOD, GSH/GSSG, and GSH-Px levels were shared with Western blot in each group.

#### 2.4.5. Experiment 5

To verify the hypothetical molecular mechanism, 24 rats (*n* = 6 per group) were assigned into 4 groups: SAH+vehicle, SAH+ACEA, SAH+ACEA+scrambled shRNA, and SAH+ACEA+Nrf1 shRNA for Western blot. Additionally, 24 rats (*n* = 6 per group) were used for neurological tests, DHE, TUNEL, immunofluorescence staining, and TEM. Samples for determination of MDA, SOD, GSH/GSSG, and GSH-Px Levels were shared with Western blot in each group.

### 2.5. Severity of SAH

The severity of SAH was estimated with the SAH grading scale as previously described [30]. Briefly, the animals were euthanized at 24 h after SAH, and the basal cistern of the rat brain was divided into 6 sections. Based on the amount of blood clotting, each section was recorded with a grade from 0 to 3. The total score of six sections ranged from 0 to 18, and rats with SAH score ≤ 8 were excluded.

### 2.6. Evaluation of Short-Term Neurofunctional Outcomes

Short-term neurofunctional outcome was estimated with the modified Garcia score and beam balance test as previously described [31, 32]. The higher score represented better neurofunctional outcome.

### 2.7. Evaluation of Long-Term Neurofunctional Outcomes

Long-term neurofunctional outcome was estimated with the rotarod test and foot fault test [33, 34]. Briefly, the rotarod test was conducted on days 7, 14, and 21 after SAH; animals were placed on a rotating horizontal cylinder. The rotating speed was started at 5 revolutions per minute (RPM) or 10 RPM and was gradually accelerated by 2 RPM every 5 seconds. The falling latency was recorded. The foot fault test was also conducted on days 7, 14, and 21 after SAH; animals were required to walk on a steel grid. A paw falling through the grid was recorded as a foot fault. A total of 50 steps were recorded for the right forelimb. Percentage of foot faults was expressed as faults/(steps + faults) × 100%.

### 2.8. Western Blot and Isolation of Mitochondria

Western blot was performed as previously described [35]. Briefly, rats were euthanized and transcardially perfused with 150 mL of cold PBS (0.01 M, pH 7.4). The left hemispheres were collected and homogenized in RIPA lysis buffer (P0013B, Beyotime, Shanghai, China). After centrifuging at 14000 × g for 30 min at 4°C, the supernatant was collected. Equal amounts of protein (30 *μ*g) were loaded onto 8%-12% SDS-PAGE gel, then electrophoresed and transferred to 0.2 *μ*m nitrocellulose membranes, which were blocked with 5% nonfat milk and incubated with the following primary antibodies overnight at 4°C: anti-CB1R (1: 1000, ab259323, Abcam, MA, USA), anti-Nrf1 (1: 2000, ab175932, Abcam, MA, USA), anti-PINK1 (1: 1000, ab186303, Abcam, MA, USA), anti-Parkin (1: 1000, ab77924, Abcam, MA, USA), anti-LC3B (1: 2000, ab192890, Abcam, MA, USA), anti-COX IV(1: 1000, ab33985, Abcam, MA, USA), anti-Bcl-XL (1: 1000, ab32370, Abcam, MA, USA), anti-Bax (1: 1000, ab32503, Abcam, MA, USA), anti-cleaved caspase-3 (1: 500, 9661, Cell Signaling Technology Inc., MA, USA), anti-Romo-1 (1: 500, NBP2-45607, NOVUS Biologicals, CO, USA), and anti-*β*-actin (1: 1000, ab8227, Abcam, MA, USA). The next day, the membranes were incubated with the corresponding second antibodies for 2 h at room temperature. Immunoblots were visualized with BeyoECL Star chemiluminescence reagent kit (Beyotime, Shanghai, China) and quantified by densitometry using the ImageJ software. COX IV and *β*-actin were used as internal control.

Mitochondrial proteins were extracted using the Tissue Mitochondria Isolation Kit (Beyotime, Shanghai, China) according to the manufacturer's instructions.

### 2.9. Immunofluorescence Staining

Immunofluorescence staining was performed as previously described [36]. Briefly, after being blocked with 5% donkey serum in 0.3% Triton X-100 for 60 min at room temperature, the brain slices were incubated at 4°C overnight with the following primary antibodies: anti-CB1R (1: 100, ab259323, Abcam, MA, USA), anti-NeuN (1: 1000, ab104224, Abcam, MA, USA), anti-GFAP (1: 50, ab4648, Abcam, MA, USA), anti-Iba1 (1: 100, ab5076, Abcam, MA, USA), anti-LC3B (1: 1000, ab192890, Abcam, MA, USA), and anti-TOMM20 (1: 100, ab56783, Abcam, MA, USA). Then, the slices were incubated with the appropriate fluorescence-conjugated secondary antibody (1: 500, Abcam, MA, USA) at 37° C for 1 h.

### 2.10. Transmission Electron Microscopy (TEM)

The morphology of mitochondria was observed by TEM. 1 mm^3^ tissue was cut from the brain of each group and fixed with 2.5% glutaraldehyde for 4 h. After dehydration, samples were embedded into araldite and then cut into 60 nm slices with an ultramicrotome (Leica, Wetzlar, Germany). At last, the slices were fixed to nickel grids after staining. Images were acquired using a transmission electron microscope (Carl Zeiss, Thornwood, NY, USA).

### 2.11. Evaluation of Oxidative Stress

#### 2.11.1. Determination of MDA, SOD, GSH/GSSG, and GSH-Px Levels

The homogenate of the left hemispherical cortex tissues was collected. The levels of cellular MDA, SOD, GSH/GSSG, and GSH-Px in cortex tissues were, respectively, detected with Lipid Peroxidation MDA Assay Kit (S0131S, Beyotime, Shanghai, China), SOD Assay Kit (S0103, Beyotime, Shanghai, China), GSH and GSSG Assay Kit (S0053, Beyotime, Shanghai, China), and Total Glutathione Peroxidase Assay Kit (S0058, Beyotime, Shanghai, China) according to the manufacturer's instructions.

#### 2.11.2. Dihydroethidium (DHE) Staining

To detect the reactive oxygen species (ROS) level of brain tissues, the brain slices were incubated with 2 *μ*mol/L DHE (Thermo Fisher Scientific, MA, USA) at 37°C for 30 min. Images were photographed by a fluorescence microscope, and the DHE-positive cells were quantified by using ImageJ software.

### 2.12. Evaluation of Neuronal Damage

#### 2.12.1. TUNEL Staining

Neuronal apoptosis was detected with TUNEL staining kit (11684795910, Roche, USA) according to the manufacturer's protocols. Briefly, after being blocked with 5% donkey serum in 0.3% Triton X-100 for 60 min at room temperature, the brain slices were incubated at 4°C overnight with the primary antibodies: anti-NeuN (1: 1000, ab104224, Abcam, MA, USA). Then, the slices were incubated with fluorescence-conjugated secondary antibody (1: 1000, ab150120, Abcam, MA, USA) for 1 h at 37° C. Lastly, the slices were incubated with TUNEL reaction mixture for 1 h at 37° C before DAPI nuclear staining. Under a fluorescence microscope, the TUNEL-positive neurons in the left temporal cortex were quantified by using ImageJ software.

#### 2.12.2. Nissl Staining

Hippocampal neuron degeneration was assessed with Nissl staining on the 28th day after SAH. Briefly, the 16 *μ*m coronal slices were incubated with 0.5% crystal violet solution for 15 min. Under an optical microscope, the Nissl-positive cells in the hippocampal cornu ammonis (CA)1, CA3, and dentate gyrus (DG) were quantified by using ImageJ software.

### 2.13. Statistical Analysis

Data were represented as mean ± standard deviation (SD) or median with interquartile range based on the normality and homogeneity of variance. For the data with a normal distribution, one-way analysis of variance (ANOVA) followed by the Tukey post hoc test was used for statistics. For nonnormally distributed data, the Kruskal-Wallis test followed by the Dunn post hoc test was used for statistics. A value of *p* < 0.05 was considered statistically significant. GraphPad Prism software and SPSS software (version 24.0) were used for statistical analyses.

## 3. Results

### 3.1. Mortality and SAH Severity

There were 33 rats in the Sham group and 209 rats in the SAH group, of which 44 rats died due to SAH induction (21.05%). None of the Sham-operated rats died, and 13 rats with SAH score ≤ 8 were excluded from this research (Supplemental Figure 2(a)). In the SAH group, blood clots were mainly distributed around the circle of Willis (Supplemental Figure 2(b)). No significant differences in SAH grade were observed among SAH groups (Supplemental Figure 2(c)).

### 3.2. Time Course Expression of CB1R, Nrf1, PINK1, Parkin, and LC3II after SAH

**Figure 1:**
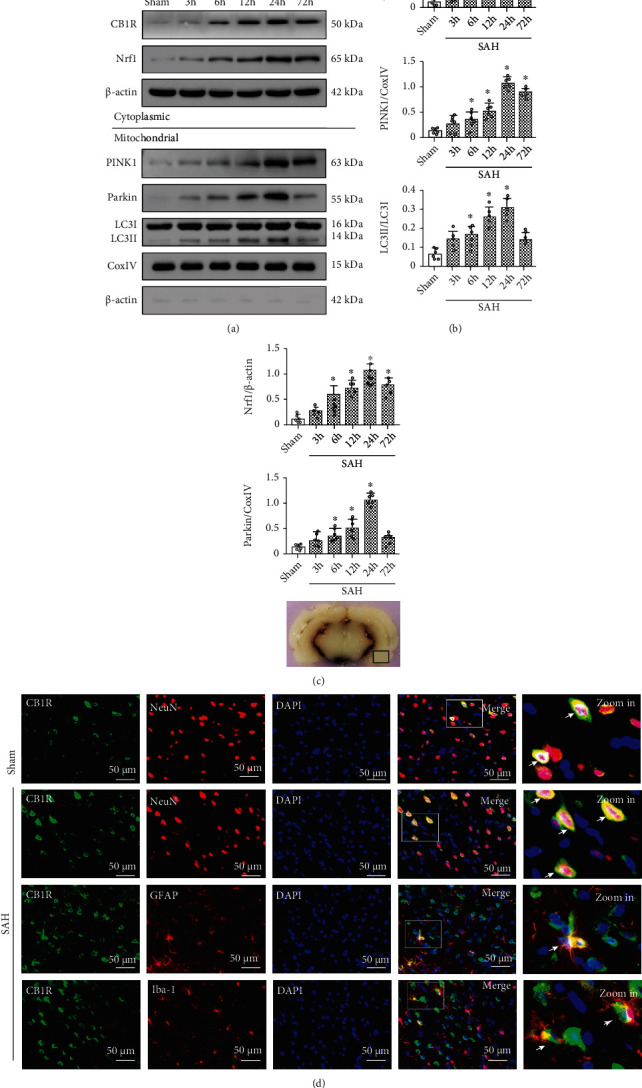
Time course expression of CB1R, Nrf1, PINK1, Parkin, and LC3II and cellular localization of CB1R after SAH. (a) Representative Western blot images of time course and (b) quantitative analyses of CB1R, Nrf1, PINK1, Parkin, and LC3II. *n* = 6 per group. Data were represented as mean ± SD. ^∗^*p* < 0.05 vs. the Sham group. (c) Representative picture indicates the location of immunofluorescence staining (small black box). (d) Representative microphotographs of immunofluorescence staining for CB1R (green) with neurons (NeuN, red), astrocytes (GFAP, red), and microglia (Iba-1, red) in the left temporal cortex at 24 h after SAH. Nuclei were stained with DAPI (blue). *n* = 3 per group. Scale bar = 50 *μ*m.

Western blot revealed that the expression of CB1R and Nrf1 in the cytoplasm and PINK1, Parkin, and LC3II in mitochondria began to increase at 6 h and reached a peak at 24 h after SAH, compared with the Sham group (*p* < 0.05; Figures 1(a) and 1(b)). Consistently, immunofluorescence staining confirmed the increased expression of CB1R after SAH. Besides, it also showed that CB1R receptors were mainly located in neurons of the cerebral cortex, but a few were located in microglia and astrocytes (Figure 1(d)).

### 3.3. ACEA Attenuated Short-Term Neurological Deficits

Modified Garcia and beam balance scores indicated that SAH contributed to significant neurological deficits, compared with the Sham group. ACEA treatment at the dose of 1.5 mg/kg significantly attenuated the neurological deficits, compared with the SAH+vehicle group (*p* < 0.05, Supplemental Figures 3(a) and 3(b)). According to neurobehavioral test results, 1.5 mg/kg was chosen as the best dosage for subsequent experiments.

### 3.4. ACEA Attenuated Long-Term Neurological Deficits and Hippocampus Neuron Degeneration

**Figure 2:**
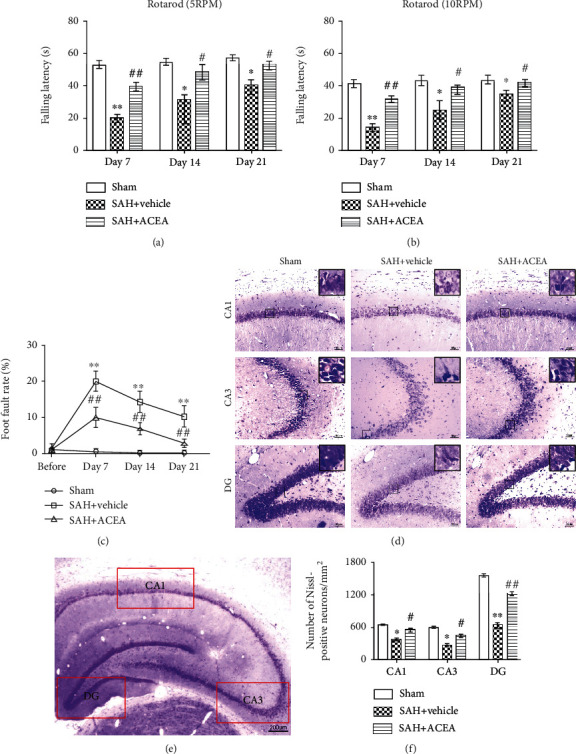
ACEA attenuated long-term neurological deficits and hippocampal neuronal degeneration after SAH. Rotarod test of 5 RPM (a) and 10 RPM (b) in the first, second, and third week after SAH, *n* = 6 per group. (c) Foot fault test during the three weeks after SAH, *n* = 6 per group. (d) Representative microphotographs of Nissl staining in the hippocampal CA1, CA3, and DG regions. Scale bar = 50 *μ*m. (e) Interest areas of the CA1, CA3, and DG region in the left hippocampus. Scale bar = 200 *μ*m. (f) Quantification of the Nissl-positive neurons, *n* = 6 per group. Data of the rotarod test were represented as the median with interquartile range. Other data were represented as mean ± SD. ^∗^*p* < 0.05 and ^∗∗^*p* < 0.01 vs. the Sham group; ^#^*p* < 0.05 and ^##^*p* < 0.01 vs. the SAH+vehicle group.

The rotarod test indicated that whether it was 5 RPM or 10 RPM, the falling latency in the SAH+vehicle group was significantly shorter than that in the Sham group. Such poor performances induced by SAH were remarkably improved by ACEA treatment (*p* < 0.05, Figures 2(a) and 2(b)).

Foot fault test showed that the foot fault rate in the SAH+vehicle group was dramatically higher than that in the Sham group in all three weeks. ACEA treatment significantly reduced the foot fault rate (*p* < 0.01, Figure 2(c)).

Nissl staining revealed that Nissl-positive neurons in the SAH+vehicle group were remarkably less than those in the Sham group in the CA1, CA3, and DG area of the ipsilateral hippocampus (*p* < 0.05, Figures 2(d) and 2(f)). ACEA treatment significantly attenuated hippocampal neuron degeneration on the 28th day after SAH (*p* < 0.05, compared with the SAH+vehicle group, Figures 2(d) and 2(f)).

### 3.5. ACEA Treatment Attenuated Neurological Deficits and Neuronal Apoptosis, whereas the Neuroprotective and Antiapoptotic Effects of ACEA Were Reversed by AM251 and Nrf1 shRNA

**Figure 3:**
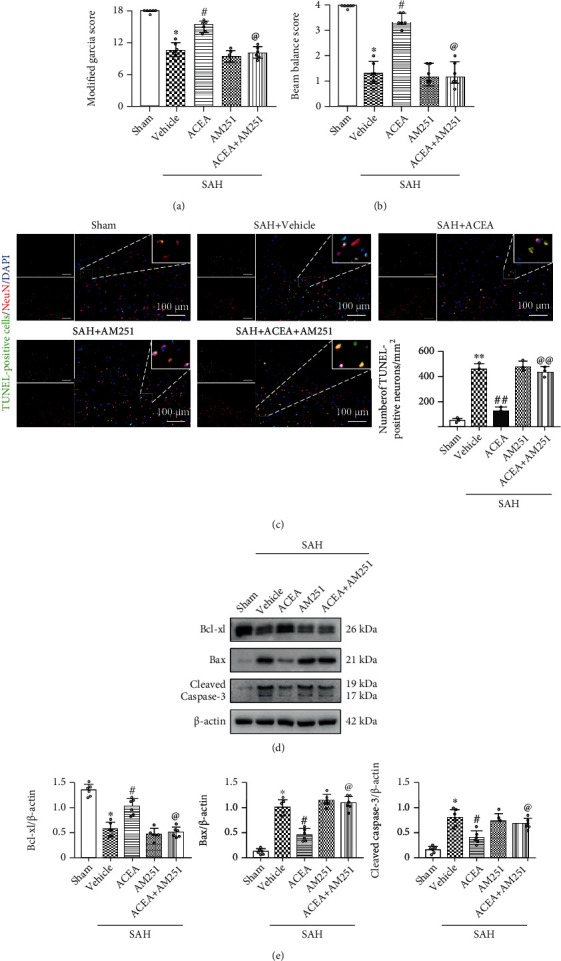
ACEA attenuated neurological deficits and neuronal apoptosis, which was reversed by AM251. (a) Modified Garcia and (b) beam balance scores, *n* = 6 per group. (c) Representative microphotographs of TUNEL staining and quantification of TUNEL-positive neurons. Scale bar = 100 *μ*m. *n* = 3 per group. (d) Representative Western blot images. (e) Quantitative analyses of Bcl-xl, Bax, and cleaved caspase-3. *n* = 6 per group. Data of Modified Garcia and beam balance scores were represented as the median with interquartile range. Other data were represented as mean ± SD. ^∗^*p* < 0.05 and ^∗∗^*p* < 0.01 vs. the Sham group; ^#^*p* < 0.05 and ^##^*p* < 0.01 vs. the SAH+vehicle group; ^@^*p* < 0.05 and ^@@^*p* < 0.01 vs. the SAH+ACEA group.

**Figure 4:**
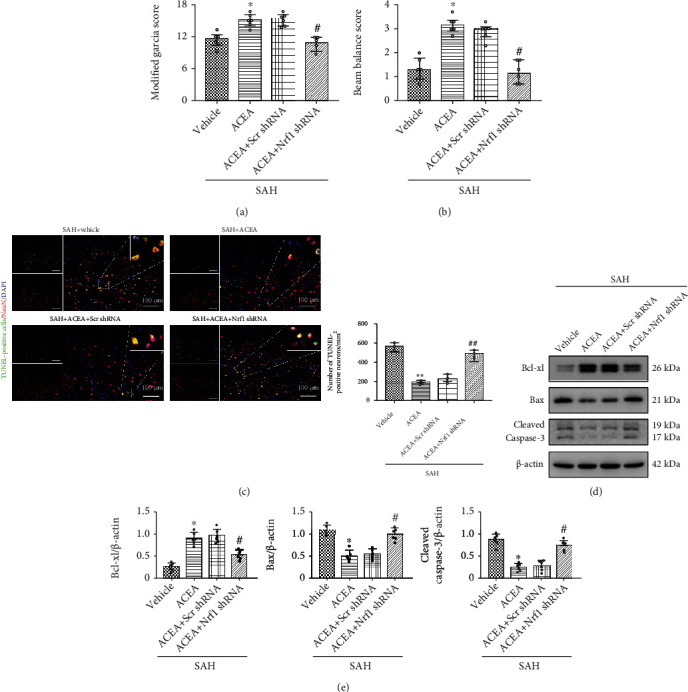
Nrf1 shRNA abolished the neuroprotective and antiapoptotic effects of ACEA. (a) Modified Garcia and (b) beam balance scores, *n* = 6 per group. (c) Representative microphotographs of TUNEL staining and quantification of TUNEL-positive neurons. Scale bar = 100 *μ*m. *n* = 3 per group. (d) Representative Western blot images. (e) Quantitative analyses of Bcl-xl, Bax, and cleaved caspase-3. *n* = 6 per group. Data of Modified Garcia and beam balance scores were represented as the median with interquartile range. Other data were represented as mean ± SD. ^∗^*p* < 0.05 and ^∗∗^*p* < 0.01 vs. the SAH+vehicle group; ^#^*p* < 0.05 and ^##^*p* < 0.01 vs. the SAH+ACEA+scrambled shRNA group.

In modified Garcia score and beam balance test results, ACEA treatment ameliorated neurological deficits compared with the SAH+vehicle group (*p* < 0.05, Figures 3(a) and 3(b)). Compared with the SAH+ACEA group, AM251 abolished the neuroprotective effect of ACEA in the SAH+ACEA+AM251 group (*p* < 0.05, Figures 3(a) and 3(b)). Compared with the SAH+ACEA+scrambled shRNA group, Nrf1 shRNA abolished the neuroprotective effect of ACEA in the SAH+ACEA+Nrf1 shRNA group (*p* < 0.05, Figures 4(a) and 4(b)).

TUNEL staining results revealed that ACEA reduced the number of apoptosis neurons compared with the SAH+vehicle group (*p* < 0.01, Figure 3(c)). Compared with the SAH+ACEA group, AM251 abolished the antiapoptotic effect of ACEA in the SAH+ACEA+AM251 group (*p* < 0.01, Figure 3(c)). Compared with the SAH+ACEA+scrambled shRNA group, Nrf1 shRNA abolished the antiapoptotic effect of ACEA in the SAH+ACEA+Nrf1 shRNA group (*p* < 0.01, Figure 4(c)).

Western blot showed that the expression of Bax and cleaved caspase-3 significantly increased, and the expression of Bcl-xl decreased in the SAH+vehicle group compared with the Sham group (*p* < 0.05, Figures 3(d) and 3(e)). AM251 eliminated the antiapoptotic effect of ACEA with the upregulation of Bax and cleaved caspase-3 and the downregulation of Bcl-xl in the SAH+ACEA+AM251 group compared with the SAH+ACEA group (*p* < 0.05, Figures 3(d) and 3(e)). Nrf1 shRNA also eliminated the antiapoptotic effect of ACEA with the upregulation of Bax and cleaved caspase-3 and the downregulation of Bcl-xl in the SAH+ACEA+Nrf1 shRNA group compared with the SAH+ACEA+scrambled shRNA group (*p* < 0.05, Figures 4(d) and 4(e)).

### 3.6. ACEA Treatment Attenuated Oxidative Stress, whereas the Antioxidative Stress Effect of ACEA Was Reversed by AM251 and Nrf1 shRNA

**Figure 5:**
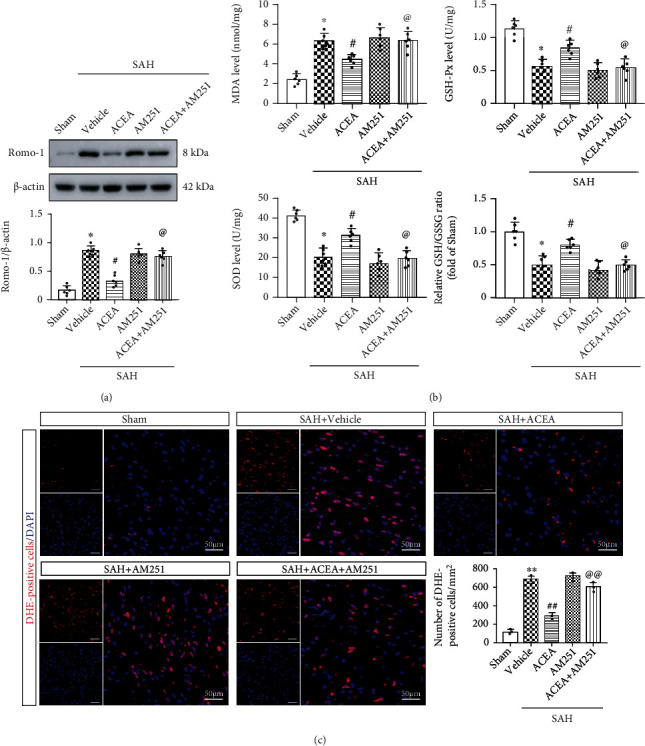
ACEA attenuated oxidative stress, which was reversed by AM251. (a) Representative Western blot images and quantitative analysis of Romo-1, *n* = 6 per group. (b) Quantification of the levels of MDA, SOD, GSH-Px, and GSH/GSSG ratio in the cortex of ipsilateral hemisphere, *n* = 6 per group (c) Representative microphotographs of DHE staining and quantification of DHE-positive cells. Scale bar = 50 *μ*m. *n* = 3 per group. Data were represented as mean ± SD. ^∗^*p* < 0.05 and ^∗∗^*p* < 0.01 vs. the Sham group; ^#^*p* < 0.05 and ^##^*p* < 0.01 vs. the SAH+vehicle group; ^@^*p* < 0.05 and ^@@^*p* < 0.01 vs. the SAH+ACEA group.

**Figure 6:**
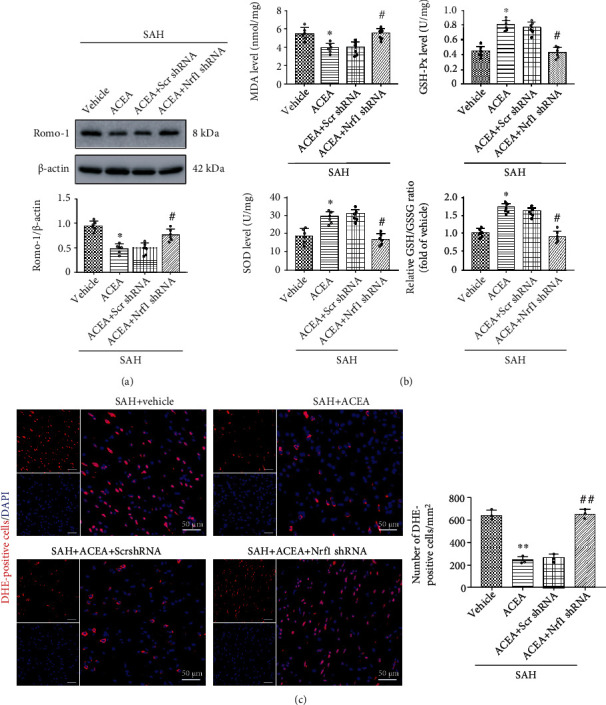
Nrf1 shRNA abolished the antioxidative stress effect of ACEA. (a) Representative Western blot images and quantitative analysis of Romo-1, *n* = 6 per group. (b) Quantification of the levels of MDA, SOD, GSH-Px, and GSH/GSSG ratio in the cortex of ipsilateral hemisphere, *n* = 6 per group (c) Representative microphotographs of DHE staining and quantification of DHE-positive cells. Scale bar = 50 *μ*m. *n* = 3 per group. Data were represented as mean ± SD. ^∗^*p* < 0.05 and ^∗∗^*p* < 0.01 vs. the SAH+vehicle group; ^#^*p* < 0.05 and ^##^*p* < 0.01 vs. the SAH+ACEA+scrambled shRNA group.

Western blot results demonstrated that Romo-1 (reactive oxygen species modulator 1), a marker of reactive oxygen species–related protein, significantly increased after SAH compared with the Sham group (*p* < 0.05, Figure 5(a)). ACEA dramatically reduced the expression of Romo-1 in the SAH+ACEA group compared with the SAH+vehicle group (*p* < 0.05, Figure 5(a)). Compared with the SAH+ACEA group, AM251 increased the expression of Romo-1 in the SAH+ACEA+AM251 group (*p* < 0.05, Figure 5(a)). Compared with the SAH+ACEA+scrambled shRNA group, Nrf1 shRNA also increased the expression of Romo-1 in the SAH+ACEA+Nrf1 shRNA group (*p* < 0.05, Figure 6(a)).

The determination of MDA, SOD, GSH/GSSG, and GSH-Px level results showed that the level of MDA dramatically increased in the SAH+vehicle group compared with the Sham group (*p* < 0.05, Figure 5(b)), whereas ACEA treatment reduced the level of MDA compared with the SAH+vehicle group (*p* < 0.05, Figure 5(b)). Additionally, the level of SOD, GSH-Px, and GSH/GSSG ratio decreased as a result of oxidative stress injury in the SAH+vehicle group compared with the Sham group (*p* < 0.05, Figure 5(b)), while ACEA treatment reinforced the activity of these antioxidative factors when compared with the SAH+vehicle group (*p* < 0.05, Figure 5(b)). However, the antioxidative effect of ACEA was, respectively, reversed by AM251 and Nrf1 shRNA (*p* < 0.05, compared with the SAH+ACEA group and SAH+ACEA+scrambled shRNA group, Figures 5(b) and 6(b)).

Moreover, ACEA treatment reduced the number of DHE-positive cells when compared with the SAH+vehicle group (*p* < 0.01, Figure 5(c)), but it was, respectively, reversed by AM251 and Nrf1 shRNA (*p* < 0.01, compared with the SAH+ACEA group and SAH+ACEA+scrambled shRNA group, Figures 5(c) and 6(c)).

### 3.7. ACEA Promoted Mitophagy and Improved Mitochondrial Morphology after SAH

**Figure 7:**
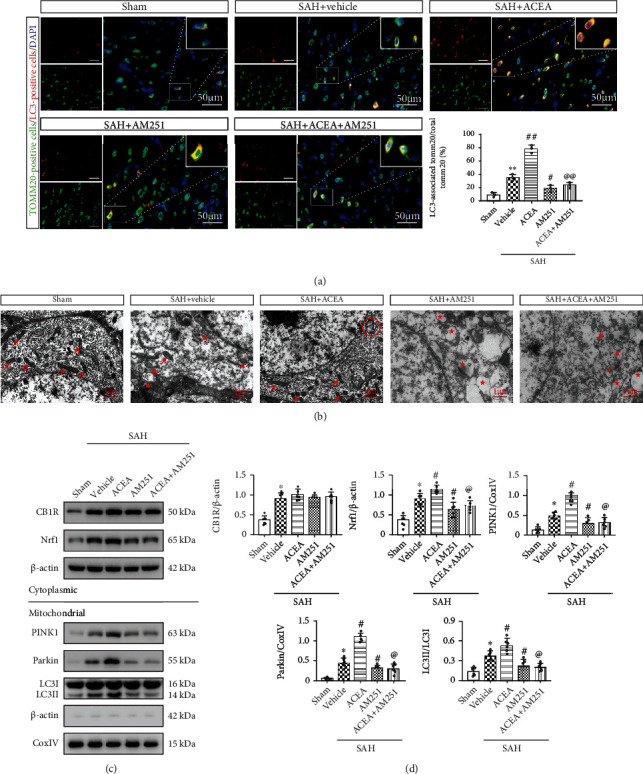
ACEA promoted mitophagy and improved mitochondrial morphology, which was reversed by AM251. (a) Representative immunofluorescence colocalization of Tomm20 (mitochondrial marker, green) with LC3 (autophagosome marker, red) and quantification of the ratio of LC3-associated Tomm20 to total Tomm20. Scale bar = 50 *μ*m. *n* = 3 per group. (b) Neuronal and mitochondrial structures were observed by TEM. Red arrow: normal mitochondria; red triangle: swollen mitochondria; red circle: mitophagosome; red star: mitochondrial vacuolization. Scale bar = 1 *μ*m. (c) Representative Western blot images. (d) Quantitative analyses of CB1R, Nrf1, PINK1, Parkin, and LC3II. *n* = 6 per group. Data were expressed as mean ± SD. ^∗^*p* < 0.05 and ^∗∗^*p* < 0.01 vs. the Sham group; ^#^*p* < 0.05 and ^##^*p* < 0.01 vs. the SAH+vehicle group; ^@^*p* < 0.05 and ^@@^*p* < 0.01 vs. the SAH+ACEA group.

In Western blot, we extracted mitochondrial proteins to detect the level of PINK1, Parkin, and LC3II in mitochondria. ACEA treatment increased the expression of PINK1, Parkin, and LC3II when compared with the SAH+vehicle group (*p* < 0.05, Figures 7(c) and 7(d)), which indicated that ACEA activated PINK1/Parkin-mediated mitophagy.

Consistently, immunofluorescence colocalization staining results confirmed that ACEA treatment increased the colocalization of mitochondrial protein TOMM20 with autophagosome marker LC3 (*p* < 0.01, compared with the SAH+vehicle group, Figure 7(a)).

Mitochondrial ultrastructural morphology was observed by transmission electron microscopy (TEM). Compared with the Sham group, there were a lot of swollen mitochondria with broken mitochondrial cristae in the SAH+vehicle group, which indicated that the mitochondrial structure of neurons had been destroyed after SAH (Figure 7(b)). ACEA treatment promoted the formation of mitophagosome and maintained the normal mitochondrial morphology of neurons (Figure 7(b)).

### 3.8. AM251 and Nrf1 shRNA Abolished the Promoting Effect of ACEA on Mitophagy

**Figure 8:**
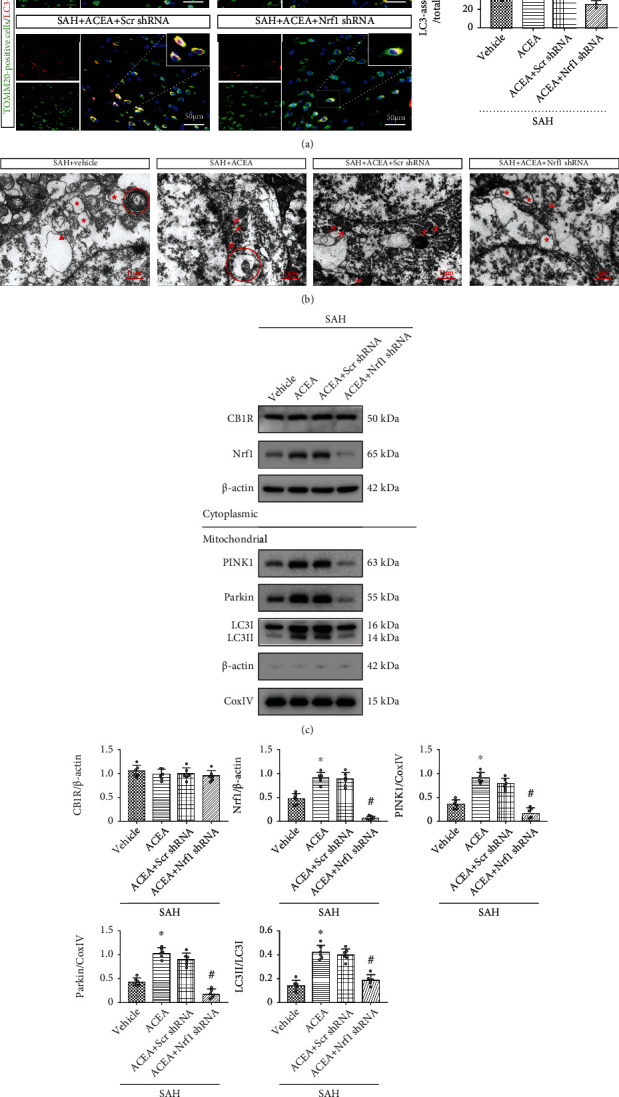
Nrf1 shRNA abolished the promoting effect of ACEA on mitophagy. (a) Representative immunofluorescence colocalization of Tomm20 (mitochondrial marker, green) with LC3 (autophagosome marker, red) and quantification of the ratio of LC3-associated Tomm20 to total Tomm20. Scale bar = 50 *μ*m. *n* = 3 per group. (b) Neuronal and mitochondrial structures were observed by TEM. Red arrow: normal mitochondria; red triangle: swollen mitochondria; red circle: mitophagosome; red star: mitochondrial vacuolization. Scale bar = 1 *μ*m. (c) Representative Western blot images. (d) Quantitative analyses of CB1R, Nrf1, PINK1, Parkin, and LC3II. *n* = 6 per group. Data were expressed as mean ± SD.^∗^*p* < 0.05 and ^∗∗^*p* < 0.01 vs. the SAH+vehicle group; ^#^*p* < 0.05 and ^##^*p* < 0.01 vs. the SAH+ACEA+scrambled shRNA group.

Western blot revealed that the expression of PINK1, Parkin, and LC3II in mitochondria significantly decreased in the SAH+ACEA+AM251 group (*p* < 0.05, compared with the SAH+ACEA group, Figures 7(c) and 7(d)), which indicated that the promoting effect of ACEA on mitophagy was eliminated by AM251. Compared with the SAH+ACEA+scrambled shRNA group, Nrf1 shRNA reduced the expression of PINK1, Parkin, and LC3II in mitochondria (*p* < 0.05, Figures 8(c) and 8(d)), which indicated that the promoting effect of ACEA on mitophagy was also eliminated by Nrf1 shRNA.

Consistently, immunofluorescence colocalization staining confirmed that the colocalization of TOMM20 and LC3 decreased in the SAH+ACEA+AM251 group (*p* < 0.01, compared with the SAH+ACEA group, Figure 7(a)). Additionally, Nrf1 shRNA also reduced the colocalization of TOMM20 and LC3 in the SAH+ACEA+Nrf1 shRNA group (*p* < 0.01, compared with the SAH+ACEA+scrambled shRNA group, Figure 8(a)).

The detection of mitochondrial morphology showed that there were many swollen mitochondria with broken mitochondrial cristae in the SAH+ACEA+AM251 group, which indicated that AM251 abolished the protective effect of ACEA on mitochondria (compared with the SAH+ACEA group, Figure 7(b)). Compared with the SAH+ACEA+scrambled shRNA group, Nrf1 shRNA resulted in significant mitochondrial destruction and even mitochondrial vacuolization in the SAH+ACEA+Nrf1 shRNA group (Figure 8(b)).

### 3.9. ACEA Promoted Mitophagy via Activation of the CB1R/Nrf1/PINK1 Signaling Pathway after SAH

Western blot demonstrated that the expression of CB1R, Nrf1, PINK1, Parkin, and LC3II markedly increased after SAH compared with the Sham group (*p* < 0.05, Figures 1(a) and 1(b)). ACEA treatment significantly increased the expression of Nrf1, PINK1, Parkin, and LC3II compared with the SAH+vehicle group (*p* < 0.05, Figures 7(c) and 7(d)). CB1R antagonist AM251 was injected at 1 h before SAH induction to evaluate whether CB1R involvement in the promoting effect of ACEA on mitophagy after SAH. The results manifested that pretreatment with AM251 remarkably reduced the expression of downstream molecules, such as Nrf1, PINK1, Parkin, and LC3II, compared with the SAH+ACEA group (*p* < 0.05, Figures 7(c) and 7(d)). The administration of Nrf1 shRNA dramatically suppressed the expression of Nrf1 in the SAH+ACEA+Nrf1 shRNA group when compared with the SAH+ACEA+scrambled shRNA group (*p* < 0.05, Figures 8(c) and 8(d)). Besides, Nrf1 knockdown significantly suppressed the expression of PINK1, Parkin, and LC3II in the SAH+ACEA+Nrf1 shRNA group when compared with the SAH+ACEA+scrambled shRNA group (*p* < 0.05, Figures 8(c) and 8(d)).

## 4. Discussion

**Figure 9:**
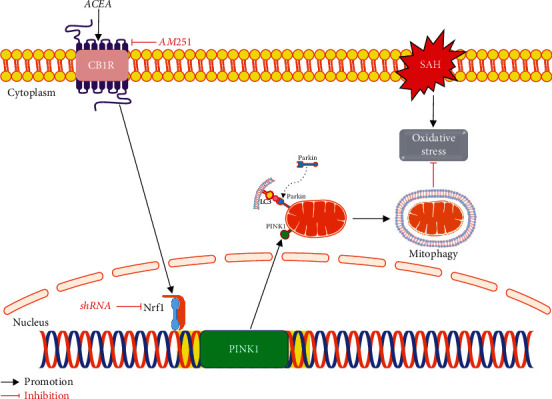
The graphical abstract. ACEA treatment attenuates oxidative stress by promoting mitophagy through the CB1R/Nrf1/PINK1 signaling pathway after SAH.

In our research, we investigated the antioxidative and antiapoptotic effects of ACEA as well as the underlying mechanism involving the CB1R/Nrf1/PINK1 signaling pathway after SAH in rats (Figure 9). We discovered that the protein level of CB1R, Nrf1, PINK1, Parkin, and LC3II began to increase at 6 h and reached a peak at 24 h after SAH. The CB1R receptors were mainly located in neurons of the cerebral cortex after SAH. ACEA treatment promoted mitophagy and exerted the antioxidative and antiapoptotic effects, which ultimately contributed to the improvement of neurological deficits. Inhibition of CB1R with AM251 eliminated the antioxidative and antiapoptotic effects of ACEA after SAH. Mechanistically, activation of CB1R upregulated the expression of Nrf1, PINK1, Parkin, LC3II, and Bcl-xl and downregulated the expression of Bax, cleaved caspase-3, and Romo-1 after SAH, whereas inhibition of CB1R reversed the above changes. Furthermore, knockdown of Nrf1 abolished the promoting effect of ACEA on mitophagy, accompanied by downregulation of PINK1, Parkin, and LC3II, which ultimately eliminated the antioxidative and antiapoptotic effects of ACEA. To sum up, our research revealed that ACEA promoted mitophagy and attenuated oxidative stress as well as neurological deficits after SAH, at least in part via the CB1R/Nrf1/PINK1 signaling pathway.

Accumulating evidence manifested that damaged mitochondria-mediated oxidative stress is closely related to the mechanism of EBI after SAH [37, 38]. Specifically, mitochondrial dysfunction caused by SAH leads to the overplus of ROS, resulting in oxidative stress injury [7] and ultimately activation of the apoptotic signaling pathway [39]. Consequently, the clearance of damaged mitochondria in time would be an efficacious way to attenuate oxidative stress as well as subsequent apoptosis after SAH [14].

Mitophagy is a selective autophagy process that specifically degrades damaged mitochondria to maintain mitochondrial homeostasis and cellular survival [8]. Recently, many studies showed that promoting mitophagy can alleviate neuroinflammation, oxidative stress, and neuronal apoptosis after SAH [11, 12, 14]. Consistent with previous studies, we found that promoting mitophagy with ACEA treatment protected against oxidative stress injury after SAH. The results of oxidative stress measurement showed that ACEA treatment contributed to an increase in SOD, GSH-Px, and GSH/GSSG levels and a decrease in MDA level after SAH. DHE and TUNEL staining results manifested that ACEA treatment reduced the number of both DHE-positive cells and TUNEL-positive neurons. Nissl staining results reflected the protective effect of ACEA on alleviating the degeneration of hippocampal neurons, which was consistent with the improvement of neurobehavior function. These results provide new insights for mitophagy as a potential therapeutic strategy for SAH.

Cannabinoid receptor 1 (CB1R), a G-protein-coupled receptor, was reported to be a potential therapeutic target for many central nervous system diseases, such as ischemic stroke, epilepsy, and Parkinson's and Alzheimer's disease [29, 40–42]. CB1R is widely expressed in different organs, especially in the central nervous system (e.g., cerebral cortex, hippocampus, striatum, and cerebellum) [43]. Our immunofluorescence colocalization staining results revealed that CB1R receptors were mainly located in neurons of the cerebral cortex, but a few were located in microglia and astrocytes. An autoptic study manifested that the expression of CB1R increased after ischemic stroke [15]. Similarly, our results demonstrated that CB1R increased to a peak at 24 h after SAH. Therefore, we speculate that the upregulation of CB1R may act as a self-protection mechanism to play a neuroprotective role in EBI after SAH.

ACEA, a highly selective CB1R agonist, was reported to provide a neuroprotective effect for ischemic stroke, Parkinson's disease, Alzheimer's disease, and epilepsy [29, 41, 42, 44]. However, there is no published research attempted to treat SAH using ACEA. In our study, we found that ACEA has the antioxidative and antiapoptotic ability to protect against EBI. In addition, we also discovered a reasonable mechanism for ACEA to improve neurological deficits after SAH in rats. Moreover, the easily passing through the blood-brain barrier characteristic of ACEA is conducive to clinical application. In order to determine the optimal dosage of ACEA, we used three different dosages in our experiment and we found 1.5 mg/kg was the best dosage to treat SAH in rats.

Nrf-1 was initially discovered as an important transcription factor that regulates genes necessary for mitochondrial function [45]. Recently, accumulating evidence indicated that NRF1 target genes are also involved in the regulation of extramitochondrial biological processes, such as DNA damage repair, RNA metabolism, and ubiquitin-mediated protein degradation, all of which are essential to cell growth, differentiation, and survival [25]. In our research, Nrf1 knockdown significantly suppressed the expression of PINK1, Parkin, and LC3II, indicating the positive regulation effect of Nrf1 on PINK1/Parkin-mediated mitophagy, which was consistent with a previous study [46].

The PINK1-Parkin pathway is a primary mechanism of mitophagy, which depends on the ubiquitination pathway. Specifically, following mitochondrial membrane depolarization, PINK1 is stabilized on the outer mitochondrial membrane (OMM). Next, PINK1 is activated via autophosphorylation and recruits E3 ubiquitin ligase Parkin from the cytoplasm to the damaged mitochondria. Subsequently, Parkin ubiquitinates multiple OMM proteins, leading to their recognition by autophagy adaptors. Finally, damaged mitochondria are engulfed by phagophores and eventually fuse with lysosomes for degradation [47]. In our research, we found that ACEA treatment increased the expression of PINK1 and Parkin in mitochondria, which indicated that ACEA might promote mitophagy through the PINK1-Parkin pathway. Consistently, immunofluorescence colocalization staining results confirmed that ACEA treatment increased the colocalization of mitochondrial protein TOMM20 with autophagosome marker LC3. The above results were further supported by transmission electron microscopy results that ACEA promoted the formation of mitophagosome and maintained the normal mitochondrial morphology of neurons. Taken together, ACEA may activate PINK1/Parkin-mediated mitophagy by upregulating the expression of Nrf1 after SAH.

In contrast to previous studies, our study is the first one that attempted to treat SAH with ACEA. We found that ACEA has the antioxidative and antiapoptotic ability to protect against EBI. More importantly, this is the first study to elucidate the molecular mechanism by which ACEA promotes mitophagy. In addition, this is the first study to confirm the positive regulation effect of Nrf1 on PINK1/Parkin-mediated mitophagy in an in vivo model of SAH. However, our study has some limitations. First, it is difficult to mimic the pathological process of SAH in vitro, so we only verified our hypothetical mechanism in vivo. Second, a previous study showed that ACEA alleviated cerebral ischemia/reperfusion injury through the CB1R-Drp1 pathway [48], so we cannot exclude other signaling pathways that improve the prognosis of SAH. These deficiencies would be explored in future studies.

## 5. Conclusion

In summary, we revealed that ACEA attenuated oxidative stress by promoting mitophagy via the CB1R/Nrf1/PINK1 signaling pathway after SAH. Therefore, ACEA may be a novel treatment for SAH patients.

## Data Availability

The data used to support the findings of this study are available from the corresponding author upon reasonable request.

## Supplementary Materials

Supplementary Materials The supplemental figures for this article can be found in the supplementary material file. Supplemental Figure 1: experimental design and animal groups. SAH: subarachnoid hemorrhage; WB: western blot; IF: immunofluorescence; TEM: transmission electron microscopy; DHE: dihydroethidium; TUNEL: terminal deoxynucleotidyl transferase-mediated dUTP nick end labeling; vehicle: 5% DMSO; ACEA: an agonist of CB1R; AM251: an antagonist of CB1R; shRNA: short hairpin ribonucleic acid; i.p.: intraperitoneal; i.c.v.: intracerebroventricular. Supplemental Figure 2: mortality and subarachnoid hemorrhage (SAH) grade. (a) Animal usage and mortality of all experiment groups. (b) Representative images of Sham and SAH groups. (c) SAH grade scores of all SAH groups. Data were represented as the medians with interquartile range. Vehicle: 5% DMSO; shRNA: short hairpin ribonucleic acid; Scr shRNA: scrambled shRNA. Supplemental Figure 3: ACEA attenuated short-term neurological deficits. (a) Modified Garcia and (b) beam balance scores, *n* = 6 per group. Data were represented as the median with interquartile range. ^∗^*p* < 0.05 vs. the Sham group; ^#^*p* < 0.05 vs. the SAH+vehicle group.
