# Supplementary material for: ACEA Attenuates Oxidative Stress by Promoting Mitophagy via CB1R/Nrf1/PINK1 Pathway after Subarachnoid Hemorrhage in Rats
Source: Oxid Med Cell Longev. 2022 Feb 24;2022:1024279. doi: 10.1155/2022/1024279 (PMC8894021; doi:10.1155/2022/1024279)
Supplement: Supplementary Materials — The supplemental figures for this article can be found in the supplementary material file. Supplemental Figure 1: experimental design and animal groups. SAH: subarachnoid hemorrhage; WB: western blot; IF: immunofluorescence; TEM: transmission electron microscopy; DHE: dihydroethidium; TUNEL: terminal deoxynucleotidyl transferase-mediated dUTP nick end labeling; vehicle: 5% DMSO; ACEA: an agonist of CB1R; AM251: an antagonist of CB1R; shRNA: short hairpin ribonucleic acid; i.p.: intraperitoneal; i.c.v.: intracerebroventricular. Supplemental Figure 2: mortality and subarachnoid hemorrhage (SAH) grade. (a) Animal usage and mortality of all experiment groups. (b) Representative images of Sham and SAH groups. (c) SAH grade scores of all SAH groups. Data were represented as the medians with interquartile range. Vehicle: 5% DMSO; shRNA: short hairpin ribonucleic acid; Scr shRNA: scrambled shRNA. Supplemental Figure 3: ACEA attenuated short-term neurological deficits. (a) Modified Garcia and (b) beam balance scores, n = 6 per group. Data were represented as the median with interquartile range. ∗p < 0.05 vs. the Sham group; #p < 0.05 vs. the SAH+vehicle group. [file 1024279.f1.doc]

# Oxidative Medicine and Cellular Longevity

**ACEA attenuates oxidative stress by promoting mitophagy via** **CB1R/Nrf1/PINK1 pathway after subarachnoid hemorrhage in rats**

Binbing Liu,^1^ Yang Tian,^1^ Yuchen Li,^1^ Pei Wu,^1^ Yongzhi Zhang,^1^ Jiaolin Zheng,^2^ and Huaizhang Shi^1^

^1^Department of Neurosurgery, The First Affiliated Hospital of Harbin Medical University, Harbin, Heilongjiang, China.
^2^Department of Neurology, The second Affiliated Hospital of Harbin Medical University, Harbin, Heilong Jiang, China.

Correspondence should be addressed to Huaizhang Shi; shihuaizhang@hrbmu.edu.cn

## Supplemental Figures


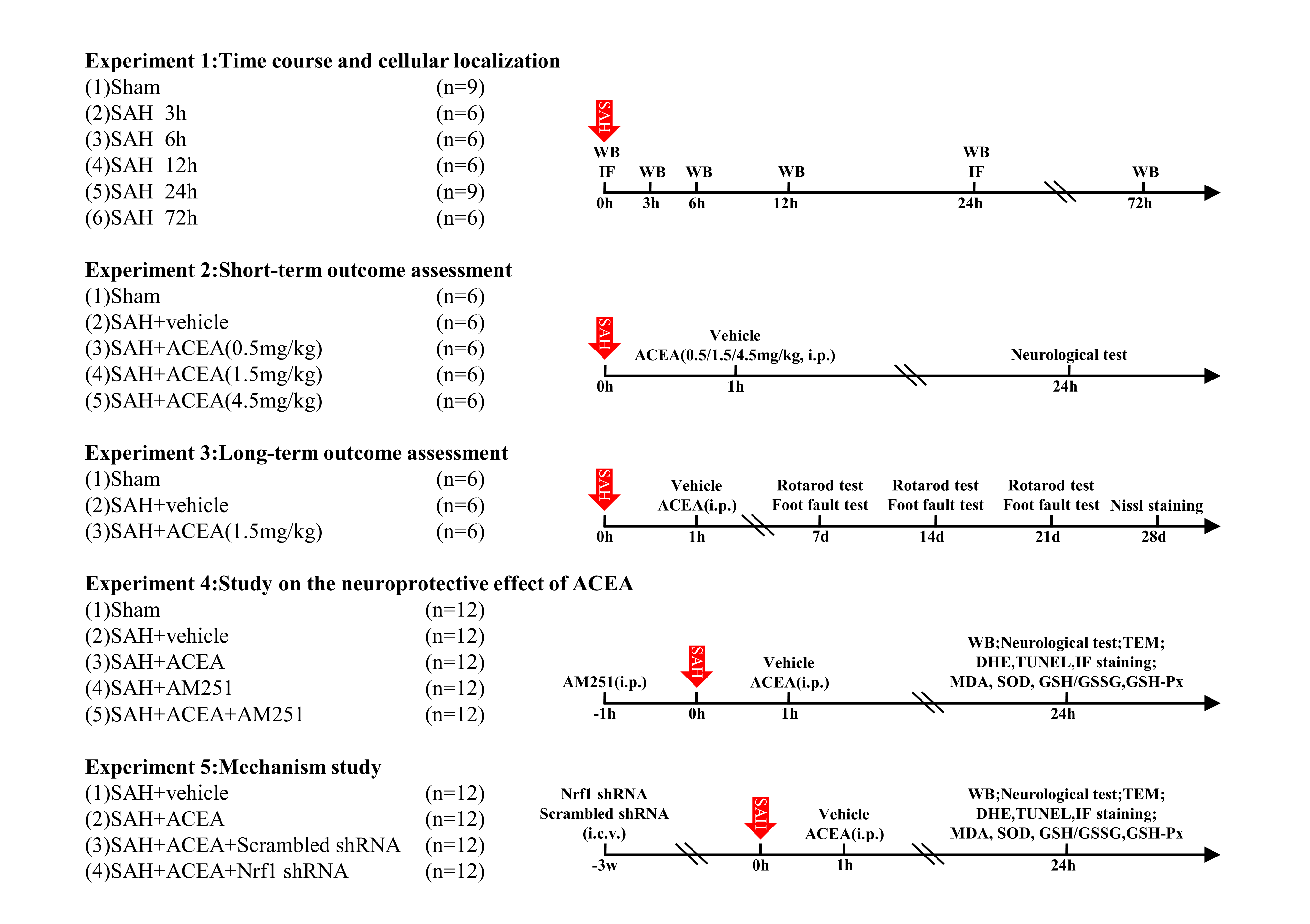


**Supplemental Figure 1:** Experimental design and animal groups. SAH: subarachnoid hemorrhage, WB: western blot, IF: immunofluorescence, TEM: transmission electron microscopy, DHE: dihydroethidium, TUNEL: terminal deoxynucleotidyl transferase-mediated dUTP nick end labeling, Vehicle: 5% DMSO,

ACEA: an agonist of CB1R, AM251: an antagonist of CB1R, shRNA: short hairpin ribonucleic acid,

i.p.: intraperitoneal, i.c.v.: intracerebroventricular.


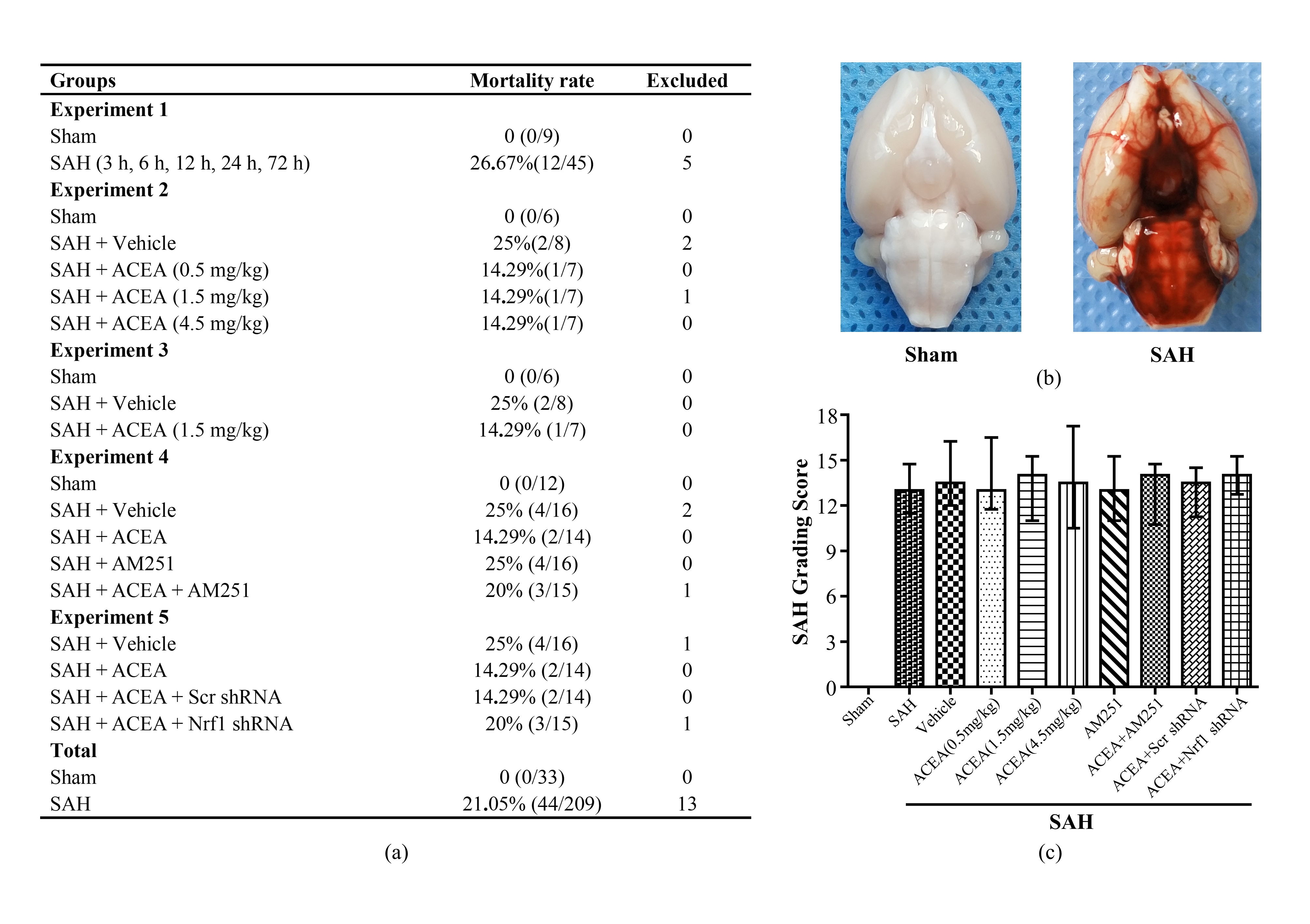


**Supplemental Figure 2:** Mortality and subarachnoid hemorrhage (SAH) grade. (a) Animal usage and mortality of all experiment groups. (b) Representative images of Sham and SAH groups. (c) SAH grade scores of all SAH groups. Data were represented as the medians with interquartile range. Vehicle, 5% DMSO; shRNA, short hairpin ribonucleic acid; Scr shRNA, scrambled shRNA.


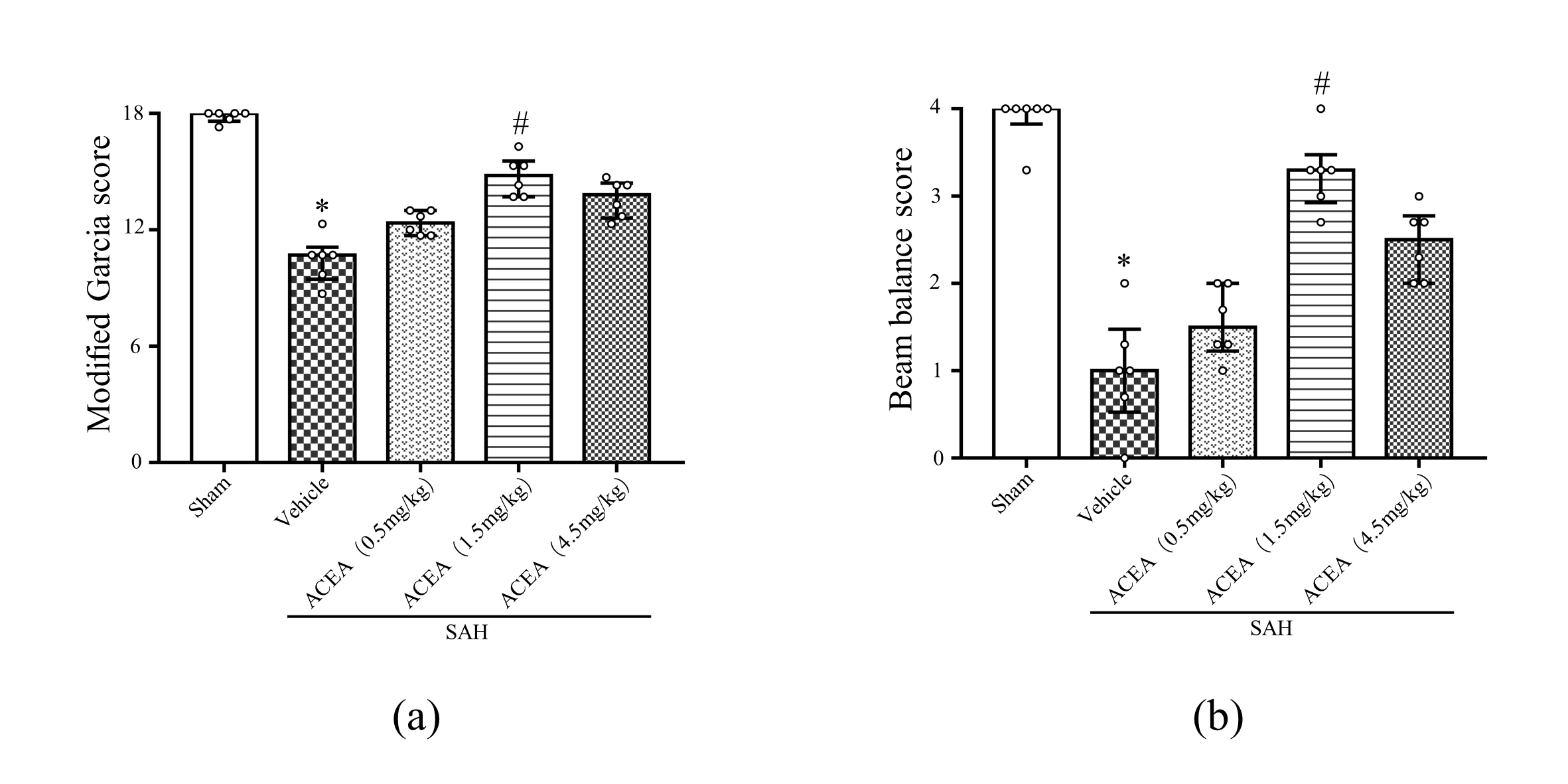


**Supplemental Figure 3:** ACEA attenuated short-term neurological deficits. (a) Modified Garcia and (b) beam balance scores, *n* = 6 per group. Data were represented as the median with interquartile range. **p* < 0.05 vs. Sham group; ^#^*p* < 0.05 vs. SAH+vehicle group.
